# Social networks and their role in preventing dementia

**Published:** 2009-01

**Authors:** Jagan A. Pillai, Joe Verghese

**Affiliations:** Department of Neurology, Albert Einstein College of Medicine, Bronx, NY, USA

**Keywords:** Cognition, dementia, elderly, social network

## Abstract

Interest in the role of social networks as a protective factor in the development of dementia over the last decade has increased with a number of longitudinal studies being published on the possible association of different lifestyles with dementia. This review examines and provides a summary of the published longitudinal studies exploring the effect of social network on dementia, with particular focus on their relevance to the Indian society. Potential cognitive and biological mechanisms mediating the effects of social networks on dementia are discussed. Results from observational studies suggest that degree of social engagement, marriage, living with someone and avoiding loneliness may have a protective effect on developing dementia that could be applicable to both Indian and western societies. A deeper analysis of the nature of social networks and dementia pertinent to Indian society is awaited.

## INTRODUCTION

Increasing life spans of populations worldwide has been accompanied by an increasing prevalence of age related diseases such as dementia. Around 25 million people worldwide have dementia, and the number of people with dementia is predicted to exceed 80 million by 2040.[1] This vast dementia burden lends a new urgency towards identifying effective preventive strategies. Social support is an important determinant of healthy aging.[2,3] Social isolation and an unengaged lifestyle have been reported to be associated with accelerated cognitive decline with aging.[4,5] The role of social environment as a risk factor for dementia has received increasing attention over the last decade.[6] This review examines published observational studies in this field with particular focus on their relevance for the Indian society.

### Healthy aging in India

In an Indian context, the joint family is often invoked as a keystone in social support and healthy aging. However, this social support system has been changing over the past few decades. Many social and demographic explanations have been proposed for changes in the traditional support systems for older adults in India. Changes in social customs in urban Indian society in recent years, the expected doubling of the elderly population by the year 2021, decrease in the number of young caregivers by declining birth rates and the out migration of children of aged parents from villages to towns and cities, and the break up of the traditional Indian joint family structure combined with the low educational and economical status of many of the elderly in the rural areas have been invoked as barriers to healthy aging in India.[7,8] The focus on the joint family structure in Indian aging studies is sometimes thought to be overemphasized with a dearth of investigations towards other possible mechanisms.[9] The role of social networks and dementia in the Indian context is therefore unclear and worthy of study. The studies being reviewed here have opened up a highly relevant question; Are some social structures more protective than others towards development of dementia and preserving cognitive function in late life? An answer to this question could have an impact on the future decisions on social and health policy of the Indian subcontinent, home to more people than Africa and South America combined.

## SOCIAL NETWORKS DEFINED

**Figure 1:**
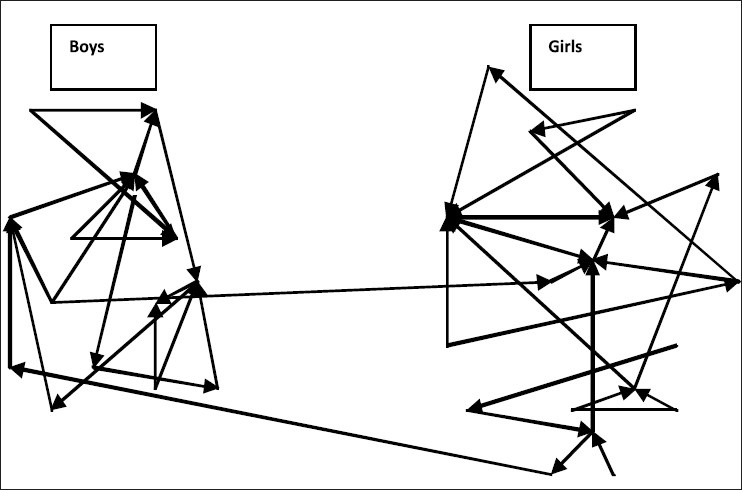
Total network of boys and girls in a classroom denoting the people they would like to sit next to (adapted from Degenne and Forse, Introducing social networks, 1st ed, Sage Publications Ltd; 1999[14])

**Figure 2:**
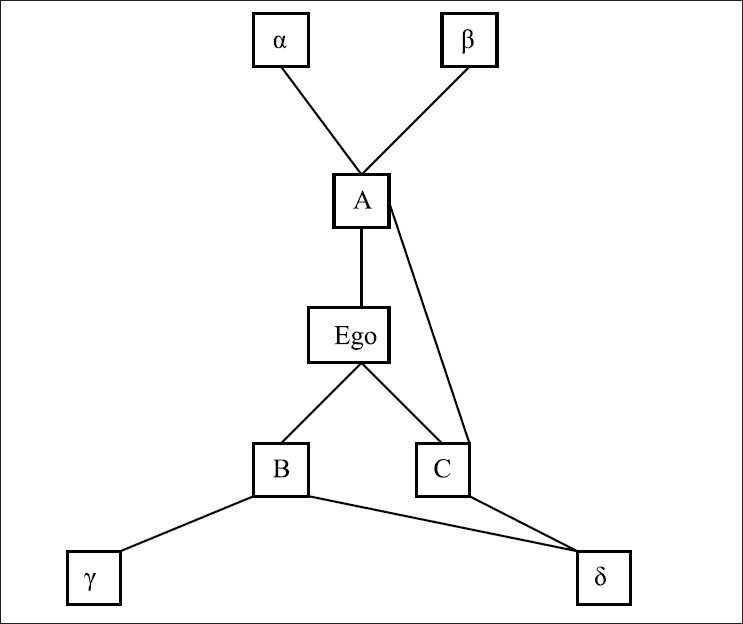
Personal network emanating from a single person (ego), total eight person network[14]

The nature of the social environment be it culture, institutions, families, groups with shared interest and/or proximity are thought to be captured by the number, frequency, and degree of interactions with other people. The term social network has been used to capture these interactions. It is defined as ‘the actual set of links of all kinds among a set of individuals’.[10] It functions as a set of relations which persons use to achieve their ends.[11] There are two approaches to the study of social networks, as the total network, such as the total number of interactions between students in a classroom [Figure 1] or as the network radiating from a single person, ‘personal social network’ [Figure 2].[12] Studies of social networks and dementia have generally used the latter approach and attempted to capture the role of ‘personal social network’ with sometimes an unstated goal of capturing social support. Most studies collected two different classes of data about social networks, the structural and quantitative aspects of social connectedness as a measure of social integration or network size and the qualitative aspects which fall under the category of social support.[6,13]

Social network, social engagement, marital status, social support, social contact, and social activities are the terms used to define social environment in the studies reviewed. The term social network is used as a descriptive term to capture the whole range of social interactions that an individual experiences. Social contact and marital status captures person-to-person interactions. Social engagement, social support, and social activities are thought to capture interactions in a social group. These descriptive classes could be overlapping by their very nature of radiating from a single person.

## CURRENT STUDIES IN SOCIAL NETWORK AND DEMENTIA

Studies of social network and dementia have looked at a number of social network variables including marital status, living arrangement, number of children, frequency of contacts and satisfaction. Close social ties has been measured by frequency of contact with friends and relatives and resultant satisfactions.[22] Social engagement has also been measured in capturing external social activities including attending religious services, going to a museum, participation in activities or groups outside home, part time or full time job.[24] One of the studies was done in a cohort of women in California[23] and another in Japanese-American men in Hawaii.[22] The rest of the studies reviewed included elderly urban populations of both sexes [Table 1] without previous diagnosis of dementia in Germany, France, Sweden, and the United States of America. The heterogeneity of reviewed studies in cognitive tests and social network measures makes precise comparison of results difficult to some extent.

Eight longitudinal cohort studies [Table 1] reported an inverse relation between the level of late-life social contacts or engagement and the risk of dementia/Alzheimer disease.[15–23] One study[24] looked at possible clinicopathological correlates of social networks measures in subjects developing dementia. It linked social networks to several measures of Alzheimer disease pathology (a global measure based on modified Bielschowsky silver stain, amyloid load, and the density of neurofibrillary tangles) with several cognitive domains. Cognitive function was inversely related to all measures of disease pathology, indicating lower function at more severe levels of pathology. Social network size modified the association between pathology and cognitive function in this study, especially for measures of semantic memory and working memory. The effects were strongest for neurofibrillary tangle pathology. Even at more severe levels of global disease pathology, cognitive function remained higher for participants with larger network sizes.[24] The authors speculated that the disease pathology modifying effects of social networks may be explained by trophic effects on brain substrates that are common to socialization and cognition, development of alternate brain networks, or that social networks could be a marker for other healthy behaviors. One longitudinal study[21] did not find midlife social engagement to be related to dementia risk but late life social engagement had a positive association to dementia. This was not consistent with the result seen in an earlier case control study[24] which revealed a significant negative association between extensive psychosocial networks even at age thirty with diagnosis of dementia decades later.

**Table 1:** Observational longitudinal studies of the association between social network and dementia[6]

Study	*n*	Age at baseline	Social network	Follow-up years	Reported associations
Bickel and Cooper *et al*.[15]	422	>65	Social relations, social support, marital status	5-8	Being single or widow with increased risk of dementia
Fabrigoule *et al*.[16]	2040	>65	Cultural, productive, and social activities	3	Traveling, odd jobs, knitting or gardening with decreased risk of dementia
Helmer *et al*.[17]	3675	>65	Marital status, social network (social ties and satisfaction), number of activities	5	Never married with increased risk of dementia and Alzheimer disease, no association with social network and leisure activities
Fratiglioni *et al*.[18]	1203	>75	Marital status, living arrangement, social ties	3	Single, living alone or no satisfying feeling with increased dementia, poor and limited social network with increased dementia
Scarmeas *et al*.[19]	1172	>65	13 selected activities (physical, cultural, recreational and social)	1-7 Mean 2.9	Single activity and factor scores (intellectual, physical and social) with decreased risk ofAlzheimer disease, higher leisure activity score with decreased risk
Wang *et al*.[20]	732	>75	Mental, social, recreational, productive and physical activities, frequency of participation	6	Frequent engagement in mental, social, and productive activities was inversely related to dementia incidence
Karp *et al*.[21]	776	>75	The leisure activities were grouped into 29 main types of activities. A mental, social, and physical component score was assigned to each of the 29 activities.	3	Having high overall scores on all 3 components was associated with significantly lower risks of dementia
					Single activities scoring high in more than one component had a substantial social component
Saczynski *et al*.[22]	222 Japanese-American men	Had follow up in both midlife and late life	Marital status, living arrangement, participation in social, political, or community groups number of face-to-face or telephone contacts with close friends per month and the existence of a confidant relationship	Midlife: average of 27.5 years before dementia diagnosis Late life: average of 4.5 years before dementia diagnosis	Lowest late-life social engagement group had a significantly higher risk of dementia Findings were similar when subtypes of dementia (Alzheimer’s disease and vascular dementia) were examined (data not shown) No association between midlife social engagement and risk of dementia
Crooks *et al*.[23]	2249 women	>78	Lubben Social Network Scale: active social network, perceived support network and perceived confidant network. Noted frequency of social contact	5	Larger social networks and daily social contact have a protective influence on cognitive function among elderly women

Only one study that we reviewed noted a lack of association between social networks and dementia[17] though it did show a positive association to marital status. Interestingly a study on cognitive decline in a Taiwanese cohort[26] showed no association between social network or support measures at home. This was despite a social structure where elderly persons often live with their children, and social interaction is likely to be more family-centered than in western countries. Data from Taiwan seems to suggest that participation in social activities outside the family may have a bigger impact on cognitive function than social contacts with family or non-relatives. These findings could arise in part as social ties can impose significant emotional demands or involve negative interactions The results may also be specific to Asian settings, where extra-family social support structures are generally thought to be strong and may not be measured adequately by the frequency of contact with specific individuals, especially family members.[23] In contrast to the previous study, no associations were seen in Chinese[27] and Canadian[28] cohorts between social activities outside the family and risk of developing cognitive decline. It is therefore still unclear how social activities vs. social support may play a role in preventing cognitive decline in different societies.

Data from longitudinal studies of social networks and dementia so far are has been consistent in showing a protective effect of social networks on dementia. There are no randomized controlled trails that have looked at the protective effect of social networks on dementia (though this may not be possible to do over a short period) and all studies so far have been from western cohorts.

## CAVEATS AND POTENTIAL MECHANISMS FOR THE COGNITIVE BENEFICIAL EFFECTS OF SOCIAL NETWORKS

### 

#### a) Cognitive reserve hypothesis

Robert Katzman proposed that individuals with higher educational levels are more resistant to the effects of dementia as a result of improved cognitive reserve due to increased neuronal synaptic complexity. A similar explanation has been proposed to explain the cognitive risk modifying effects of social networks.[24]

A significant burden of dementia in the world is vascular dementia.[29] Vascular risk factors are directly involved in the pathogenesis and progression of these dementias in addition to their involvement in Alzheimer’s dementia.[30] Cerebrovascular disorders could also promote earlier clinical expression of dementias by reducing cognitive reserve. Social, mental, and physical activities could act via their positive effects on the cardiovascular system and help prevent the progression of dementias.

#### b) Social networks as a marker for healthier lifestyle

Neurobiological mechanisms underlying these epidemiological effects are unknown. Subjects with more social engagement could also be self-selecting, with high-ability individuals leading intellectually and socially active lives, which allows the possibilities that residual confounding (the effect of an unmeasured factor) and/or reverse causality (incipient decline affecting the seeking of cognitive stimulation) may explain these associations.[31] A life course approach to cognitive function would postulate that both biological and psychosocial factors operating in early life may be important.[32] Social network could be a marker for early life protective factors such as education or it may help maintain the cognitive benefits of education later in life as adjustment for the psychosocial network neutralized the otherwise protective effect of education for dementia of any type and for possible vascular dementia in a case control study.[25] Those with larger social networks are also thought be less depressed which also is associated with cognitive decline and dementia. However, the association of social networks with reduced risk of dementia shown in recent studies remains significant even after these confounding effects are taken into consideration.[6,24]

#### c) Cultural factors influencing social networks and health

A conceptual model of cascading casual process beginning with the larger social and cultural context that determines the social network structure and characteristics of network ties has been proposed.[5] Social networks may affect health by operating through five main mechanisms; social support, social influence, social engagement, person-to-person contact and access to material resources and goods which affect health through behavioral, psychological and physiological pathways. There is now an increasing appreciation of psychological and behavioral intermediate mechanisms through which social networks may affect final biological pathways by disease modification.[33]

Persons living in joint families have larger social networks than in nuclear families. Significant network size differences were shown between joint family males and nuclear family males and females, a patriarchal joint family having a positive impact on aging in terms of gender. Being an older male in a joint family results in high social involvement.[7] This shows the importance of understanding the social roles and their embededness[34] in the social context as it can lead to differing amount of social involvement. The type of family structure, the number of social interactions and the social role these interactions play are unanswered in these present studies. Some studies on cognitive aging[33] have shown that the number of social relationships do not affect cognitive decline as much as the quality of relationships. The quality of relationships and their role in development of dementia has yet to be investigated. These could be captured by variables like changes in the economic and social roles, degree of personal vs. nonpersonal contact (e.g.: use of telephone, internet, etc). These questions also make the cultural context of the recent findings on dementia and social network relevant to keep in mind.

#### c) Neurobiological aspects of social cognition

Social cognition is defined as the perception of others, the perception of self and interpersonal knowledge. The basic cognitive processes in social cognition involve the perception, a social stimulus (the self, other people or the interaction of the two) in varying degrees of complexity. Later stages of elaboration integrate basic perceptions with contextual knowledge and finally involve representations of possible responses to the situation.[35] A highly social animal engages many perceptual and cognitive systems including vision, audition, speed of processing, episodic memory and attribution of mental states.[36,37] Deficits in any of these systems that are commonly seen with age could lead to poor social functioning and confound the effects of social networks in the development of dementia. This makes the involvement of a single social module that is more vulnerable in dementia less likely.

In the neuroscience literature two sets of findings, one at a macroscopic level, the other at a microscopic level suggest that the primate brain might contain neural systems specialized for processing socially relevant information. Selective lesions of the monkey amygdala result in more subtle impairments which appear to impair disproportionately impact those behaviors normally elicited by social cues with abnormal emotional reactivity in social situations being a common occurrence.[38] At the level of single neurons, neurophysiological studies in non-human primates have shown that single neurons in the monkey inferotemporal cortex respond relatively selectively to the sight of faces, modulate their response preferentially with specific information about faces, such as their identity, social status or emotional expression and are modulated by viewing complex scenes of social interaction and specific features of faces that can signal social information, such as gaze direction.[38,39]

Several developmental disorders including autism spectrum disorders, schizophrenia, behavioral variant frontotemporal dementia (FTD) and Parkinson’s disease have impaired ability to maintain social ties.[39,41] Studies converge on a set of neural structures that are presumed to mediate our perception and interpretation of the social meaning of other people. These include higher-order visual cortices in the temporal lobe, amygdale and orbitofrontal cortex, and additional cortical regions, such as the left prefrontal and right parietal cortices, these have been implicated in maintaining self vs. non-self thoughts, feelings and other aspects of normal social functioning including episodic and semantic memory.[38] Damage to these areas by progressive degenerative disorders or neurodevelopmental abnormalities are thought to mediate poor social functioning in these conditions. In contrast to FTD, Alzheimer disease often spares social functioning until late in its course, damaging instead a posterior hippocampal-cingulo temporal-parietal network involved in episodic memory retrieval.[42] The present epidemiological work on the role of social networks and dementia do not always take the varieties of pathological variations seen in dementia like Alzheimer dementia, vascular dementia, and frontotemporal dementia into consideration.

In animal studies, environmental complexity and richness (argued to be the equivalent of a rich social environment in a human context) prevent cognitive decline,[43] decrease amyloid load,[44] and promote neurogenesis.[45] Enriched transgenic mice expressing amyloid precursor protein (APP), presenilin1 (PS1) and APP X PS1 perform significantly better in cognitive tests than their standard-housed counterparts after five to seven months of enrichment housing and maintain the same level of performance as wild-type controls kept in standard cages.[46] In the animal models of neurodegenerative diseases it is still unclear at what time period from dementia the interventions are most effective or if they are protective towards development of dementia.

#### d) Social networks as a stress reduction mechanism

Active individuals with more frequent contacts and integration have more opportunities for social engagement and positive emotional states. This could potentially lead to lower stress, though there is a counter argument to be made for the role of stressful relationships in society. The stress response and levels of associated hormones, which have been linked to brain function,[2] have been postulated to be modified by social engagement.

Over weeks, months, or years, exposure to increased secretion of stress hormones can result in allostatic load and its pathophysiologic consequences. Most common allostatic responses involve the sympathetic nervous systems and the hypothalamic-pituitary-adrenal axis.[47] Aged subjects showing a significant increase in cortisol levels with years and with high current basal cortisol levels were impaired on tasks measuring explicit memory and selective attention when compared to aged subjects presenting either decreasing cortisol levels with years or increasing cortisol levels with moderate current basal cortisol levels.[48] Assessments of declarative and spatial memory have shown individual differences in brain aging concomitant with atrophy of the hippocampus and progressive elevation of cortisol.[49] Social interactions have been shown as having stress buffering property in the aged and improving functional outcome.[50]

## UNANSWERED QUESTIONS FROM THE STUDIES ON SOCIAL NETWORKS AND DEMENTIA

The most pressing question that still remains unanswered from these studies is their lack of demonstration of causality. As noted earlier the association of social networks and dementia could also be a non-causal association. One way to get to the point of causality indirectly could be to investigate the rate of change of social networks. Do different rates of decrease in social networks all equally predict development of dementia or if a higher rate of decrease in social networks predicts dementia to be more likely in the future in an individual? It could be argued that social networks are a strong predictor of development of dementia in the latter case.

Social withdrawal could also be an effect of prodromal or mild cognitive deficits. In which case the protective effect of social networks would then be just an epiphenomenon and would be falsely implicated towards modifying the rate of progression of the neurodegenerative or compensatory process themselves. This issue can be addressed by making better cognitive or biological markers of mild cognitive deficits than are available now and would be an area of progress in the coming years. Studies on birth cohorts as they mature will also be crucial in being able to test this model more directly by using longitudinal measures of cognitive performance measured both in childhood and adulthood.[51]

The role of personality and a lifetime of learning through social interaction and the role people see themselves as playing as they age are very different and provide varying underlying themes to many choices and life styles people have as they age. Compared with both extroversion and introversion, moderate extroversion was associated with lower risk of cognitive impairment in both case-control and co-twin designs.[52] These factors build different levels of social engagement in a person but the studies so far have not parsed the effect of these factors in the development of dementia.

Midlife social engagement was assessed by one of the longitudinal studies[22] but was not found to be related to dementia risk. There remains the question, if intervention later in life by richer social interactions via creating synthetic social networks to either augment or substitute for naturally occurring networks can alter the onset and progression of dementia.

## CONCLUSIONS

The present array of studies have started to peel back the many layers of social functioning that could affect the development of the final biological effect of dementia. This could help eventually set up better social and health polices and practices to ensure a better quality of engaged life in society with aging. An important unanswered question relates to causality, does social network have a protective role in reducing the risk of developing dementia or could the observed association result from people less likely to have dementia being more likely to maintain their social networks over others. This is a crucial question to be answered before the findings of these previous studies can be found to have a wider applicability. Though factors such as a rural residency have been shown to influence prevalence of dementia in some societies,[53] little research has explored the role of social networks in a rural cohort as a predictor of dementia. Despite the many caveats that exist in transferring these results to an Indian context, where the majority of its citizens still live in a rural setting, results in terms of a crude binary response of absence or presence of social engagement, marriage, living with someone and avoiding loneliness[6,24] could be argued to be more applicable to both Indian and western societies. A deeper analysis of the nature of social networks pertinent to the structure of Indian society is awaited. Defining the many interlacing effects of social and environmental factors that envelop an aging individual and are peculiar to India, will help develop supportive social policies and guide cultural drift to a healthier future in aging.
